# What can transaction costs tell us about governance in the delivery of large scale HIV prevention programmes in southern India?

**DOI:** 10.1016/j.socscimed.2011.01.019

**Published:** 2011-06

**Authors:** Lorna Guinness

**Affiliations:** London School of Hygiene and Tropical Medicine, Public Health and Policy, Keppel Street, London WC1E 7HT, United Kingdom

**Keywords:** HIV, Transaction costs, Contracting out, India, Asia, Governance, NGO

## Abstract

This paper aims to understand the transaction costs implications of two different governance modes for large scale contracting of HIV prevention services to non-governmental organisations (NGOs) in 2 states in India as part of the National AIDS Control Programme between 2001 and 2003.

Interviews at purposively selected case study NGOs, contracting agencies and key informants as well as document review were used to compile qualitative data and make comparisons between the states on five themes theoretically proposed to shape transaction costs: institutional environment, informational problems, opportunism, scale of activity and asset specificity (the degree to which investments made specifically for the contract have value elsewhere).

The State AIDS Control Society (SACS) in state Y used a management agency to manage the NGO contracts whereas the SACS in state X contracted directly with the NGOs. A high level of uncertainty, endemic corruption and weak information systems served to weaken the contractual relationships in both states. The management agency in state Y enabled the development of a strong NGO network, greater transparency and control over corrupt practises than the contract model in state X. State X’s contractual process was further weakened by inadequate human resources.

The application of the transaction cost framework to contracting out public services to NGOs identified the key costs associated with the governance of HIV prevention services through NGO contracts in India. A more successful form of relational contract evolved within the network of the contract management agency and the NGOs. This led to improved flows of information and perceived quality, and limited corrupt practises. It is unlikely that the SACS on its own, with broader responsibilities and limited autonomy can achieve the same ends. The management agency approach therefore appears to be both transaction cost reducing and better able to cope with the large scale of these contracting programmes.

## Highlights

► Qualitative study of governance of NGO contracts for HIV prevention services. ► Transaction cost analysis using case studies in two Indian states. ► Informational problems leave contracts vulnerable to opportunism. ► Relational contracts evolve in both settings. ► More successful contracting achieved when state recruits contract management agency.

## Introduction

At the start of the 21st century, India had put in place a massive scale up of HIV prevention programmes. With the support of the World Bank over 200 million USD was allocated to a five year AIDS control programme. The programme focussed on expanding the most cost-effective group of HIV prevention services, given the early stage of the epidemic (National AIDS Control Organisation, 2000, 2001; World Bank Health Nutrition and Population Sector Unit, 1999). Approximately one quarter of the budget was to be spent on targeted interventions (HIV prevention for vulnerable groups) and strengthening services for sexually transmitted infections (STIs) (National AIDS Control Organisation, 2000). Given the lack of capacity within existing health services and building on positive experience from Tamil Nadu (Ramasundaram, Allaudin, Charles, Gopal, Krishnamurthy, Poornalingam et al., 2001), services for vulnerable groups were contracted out to NGOs by a semi-autonomous government agency that operated at the state level – the State AIDS Control Society (SACS). Contracting out had the multiple aims of increasing coverage, avoiding inefficiencies of government bureaucracy and taking advantage of the assumed efficiencies of NGOs.

Contracting out health services in developing countries has been used as a tool for increasing coverage and quality and improving efficiency and equity (Liu, Hotchkiss, & Bose, 2008; Loevinsohn & Harding, 2005). Contracting out covers a range of activities, including the development of franchise networks, partnering with private sector providers for TB treatment and contracting with private providers to provide health services. Experience to date has shown these efforts can be an important leverage for increasing coverage (Liu et al., 2008; Loevinsohn & Harding, 2005). Improvements in quality have also been observed as a direct result of contract design in the form of performance based financing (Liu et al., 2008; Loevinsohn & Harding, 2005). However these studies tell us little about the effectiveness of contract design and the costs of the governance arrangements. Evaluation of the experience of contracting out in low income countries has largely focused on the final outcomes of the health services. And even where increases in coverage have been observed, little is known about the quality of the service delivery (Liu et al., 2008). Insights into the nature of the contracts and how they ultimately influence provider performance are key to help improve contractual design (Palmer & Mills, 2005). Two papers are notable exceptions in the literature that explore the institutional and environmental context in which contracting out operates in low and middle income countries (Palmer & Mills, 2005; Palmer, Strong, Wali, & Sondorp, 2006).

Transaction cost economics helps to deconstruct the contractual process by exploring informational problems, rent-seeking and the issue of asset specificity – i.e. the cost of giving up the contract due to investments made that relate specifically to the contract and have little value elsewhere (Allen, 2002; Ashton, 1998; Goddard & Mannion, 1998; Marini & Street, 2007). Transaction costs are defined as the costs of negotiating, establishing, safeguarding and enforcing contractual agreements (Williamson, 1983). They and the associated governance are shaped by the presence or not of bounded rationality (or informational problems) and uncertainty; opportunism and asset specificity (the degree to which investments can be re-allocated to other uses if the contract is terminated). The frequency of the transaction and the institutional environment also serve to further influence the transaction costs (Allen, 2002; Ashton, 1998; Palmer, 2000; Williamson, 1983, 1996). In theory, the inter-play of all these factors lead to costs, or expected costs, that result in decisions regarding the appropriate governance form. These can range from: markets in which separate organisations or individuals contract directly with each other and all information pertaining to the contract is known by all parties in the transaction; to hierarchies, e.g. bureaucracy defined as a “nexus of contracts” governed by fiat rather than contract law and generally characterized by low powered incentives (Alchian & Demsetz, 1972; Williamson, 1991). In between these polar extremes, hybrid forms of governance exist where bilateral dependence has evolved between independent organizations. One such form is Macneil’s relational contract, in which, in the absence of a fully specified contract, an ongoing relationship leads to self-regulation and development of a set of behavioural norms (Hviid, 2000).

Traditionally applied to the for profit sector where governance decisions are based on the profit maximising motive of firms, transaction cost analysis can also provide useful insights into the operation of a contractual process where governance structures are pre-determined such as in the public sector (Palmer & Mills, 2003). In New Zealand and South Africa respectively, Ashton (1998) and Palmer and Mills (2005) showed how the nature of the contract varies with the types and ranges of services (Ashton, 1998; Palmer & Mills, 2005). Studies of contracting in the English NHS and South Africa have indicated the importance of relational contracts and long term flexible contracts in a sector that is unpredictable, heterogeneous and dynamic, and where providers are scarce (Allen, 2002; Forder, Robinson, & Hardy, 2005; Goddard & Mannion, 1998; Palmer & Mills, 2005).

Applying this framework to the case of HIV prevention in India, there are five key factors likely to affect the transaction costs and governance of contracts. First, as for any contractual process, the institutional environment that regulates the contract needs to function effectively, whether this is the legal system itself or the bureaucracy’s own form of governance. Second, informational problems exist in both specifying contracts for HIV prevention projects and monitoring. HIV prevention projects for vulnerable groups are based on making regular contacts with the target population, building a rapport with that population and, during interactions, providing quality sexual health education, condoms and referral to partner doctors, for management of sexually transmitted infections. The population of concern frequently moves locations, temporarily or permanently, to find their best market, are generally excluded from society and often kept out of reach by their madams or pimps. The multiple objectives (to provide education, improve health of the individuals and prevent HIV in the community) that are dependent on the building of community networks, the mobility of the population and the complex nature of the link between behaviour change and HIV transmission mean that contracts cannot be fully specified but pertain only to general areas of activity that have been shown to be effective in HIV prevention. Monitoring the contract requires information regarding the population size, location, number of times an individual has been reached and the quality of interactions. The dynamic nature of the population and the sensitivity of the issue of HIV make monitoring of the quality and quantity of interactions difficult. This is compounded by the limited capacity for monitoring both among the SACS and the NGO staff and volunteers. As the NGO has built the links that enable access to the community, independent verification of project activity is also difficult to achieve. A supplementary method to monitor progress would be to measure impact on the spread of HIV. However, data on the impact of HIV prevention interventions are rarely available as generation of such impact data requires expensive epidemiological trials or mathematical modelling (Grassly, Garnett, Schwartlander, Gregson, & Anderson, 2002; Merson, Dayton, & O’Reilly, 2000). Even if these data were available the impact would be difficult to attribute to a specific project given the dynamics of the target populations and their sexual partners and the possible presence of other HIV prevention activities in the same locality.

The third factor, opportunism, in the form of corruption is endemic in India (Transparency International, 2002). Fourth, asset specific investments are likely to be present in two forms. Human asset specificity is associated with working in HIV/AIDS and sexual health and with commercial sex workers. Project staff need both the ability to overlook society’s disapproval of commercial sex workers and those connected with them, as well as skills and experience in connecting with a vulnerable community and counselling on sexual health topics. The need for local NGOs and their partners that are established within their communities also implies “*temporal asset specificity*” in which on-site human assets are required in the delivery of the project (Williamson, 1991). These assets would include doctors willing and able to work with commercial sex workers on sexual health, peer educators and others with knowledge of the local sex worker community and its networks. Finally, large numbers of contracts may compound the information problems. According to the theory, the combination of asset specificity, opportunism and informational problems can be exacerbated in a situation of large numbers and will result in what Williamson terms “*serious contractual difficulties*” where more hierarchical forms of governance are likely to evolve to minimise transaction costs (Williamson, 1987).

In order to better understand the contractual processes and key factors in their successes and failures, this paper explores the transaction costs in the contractual relationships between NGOs and the state in the delivery of HIV prevention programmes for vulnerable groups in southern India. Using qualitative methods, it compares the nature of transaction costs of two different contracting models - the SACS programmes of State X and State Y. In so doing it attempts to test the theoretical proposition that, in the case of publicly financed health services, although behaviours are not necessarily aligned with profit-maximisation, more hierarchical forms of governance are still better able to cope with serious contractual difficulties and the complexities of contracting with large numbers.

## Methods

### Sample

**Fig. 1:**
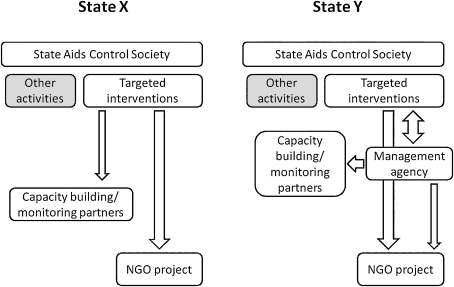
Models used for contracting out HIV prevention services to NGOs in State X and State Y.

Two Indian states with differing modes of NGO contracting, that were contracting with more than 50 NGOs and where adult HIV prevalence was considered to be high (1% or higher) at the time of data collection (2002) were selected. The government supported AIDS control programmes in State Y and State X were implemented by 3 SACS. XSACS, XMSACS were in State X and YSACS in State Y. Each of these agencies were responsible for the range of HIV/AIDS activities within their state or municipal region including the contracting out to NGOs the delivery of HIV prevention services for targeted populations, such as commercial sex workers (CSWs), truck drivers, slum dwellers and men who have sex with men (MSM). XSACS and XMSACS contracted directly with the NGOs whereas YSACS employed an intermediary management agency to manage the contracting process, from tendering to capacity support to monitoring and evaluation. The two contracting models are outlined in Fig. 1.

For each contracting model, a case study approach was used to collect data on the transaction costs of contracting. Commercial sex worker (CSW) projects were selected from the range of different projects, to ensure like was compared with like and so strengthen comparative analyses. In state Y, case studies were selected to represent a range of project history, levels of capacity and experience and location. At XSACS interviews were held with those NGOs available and willing to participate, as many of the NGOs initially selected using the former criteria did not respond to our invitation. At XMSACS, the only 2 CSW projects functioning at the time of our census were selected. In addition, to see how transaction costs varied across projects for other target groups, 2 non CSW projects were included in the analysis. The case study numbers and descriptions are summarised in Table 1.

### Data collection and analysis

The non-explicit nature of some transaction costs and the need to understand the transaction characteristics, including patterns of institutional behaviour and the institutional environment, mean that quantitative analyses alone fail to capture the transaction cost problem. As a result, qualitative research methods using purposively selected case studies to represent and describe different contracting models, semi-structured interviews and a survey of documents were applied to gain a greater understanding of transaction costs (Allen, 2002; Ashton, 1998; Masten, Meehan, & Snyder, 1991; Palmer & Mills, 2003). The analysis used a deductive approach to examine whether the governance implications of variations in transaction cost patterns suggested by Williamson (1983) hold for contracting out HIV prevention services (Williamson, 1983). Following Mays and Pope (2000), the paper assumes a “*subtle realist*” approach i.e. that in spite of the subjectivity of research, there is some underlying reality, independent of the researcher, that can be identified through the research process (Fulop, Allen, Clarke, & Black, 2001; Mays & Pope, 2000). The data were collected between 2002 and 2004 and anonymised in order to encourage participants to talk freely.

The semi-structured interviews were carried out between December 2002 and May 2003 by the author (a non-Indian female, with no local language knowledge) in the presence of a research assistant from the state in which the project was located, following an interview guide. They were carried out with the directors, finance managers and those responsible for the management of the HIV prevention projects at the SACS and management agencies (referred to as XSACS or YSACS), project directors and project managers at the NGOs and a set of key informants (KI). Key informants were identified during the process of data collection and included those individuals found to have extensive either current or historical knowledge of one or all the HIV prevention programmes. Interviews were conducted in English. English language ability was sufficient that it was not necessary for any of the interviews to be wholly conducted in a local language. If a problem of language arose, the research assistant translated questions and responses. Where permission was granted, interviews were taped and transcribed by the author. In all cases, notes were taken by both the author and research assistant and cross-checked against each other and the transcriptions, where available, for consistency. Any issues that needed clarification were followed up with further interviews.

The transaction costs of the contracting out of services to NGOs were analysed by examining five areas of influence: the institutional environment; informational problems including bounded rationality and uncertainty; opportunism; and asset specificity. To contextualize these four areas and to better understand the impact of the scale of the programme, the contractual process including the size of each programmes are first described. Evidence of these different aspects of transaction costs was extracted from interview transcripts and documents and the qualitative data were then manually coded according to those themes. Important themes were identified through triangulation of data, in the form of issues raised by more than one source. Where data sources appeared to contradict the theory or each other, these contradictions were reassessed in light of the context of the data source. The transaction cost implications were classified according to whether they were “direct” and would be incurred through monitoring and management or indirect and incurred through wastage, leakage or poor quality. Comparisons of the themes were then made across the contracting models. A first draft of the findings was circulated around the research team and two key informants, who agreed to read the document, for review and corroboration.

## Results

### The contractual process

The general contractual process that each SAC adhered follows guidelines issued by the National AIDS Control Organisation (NACO) in Delhi. The SACS contracted with the NGO to provide HIV prevention services for vulnerable groups having selected the NGOs through a competitive tendering process. Project guidelines meant that each NGO project comprised four basic components: condom distribution; referral for STI treatment or treatment itself; educational activities (one-to-one and group); and strengthening links with the community. Once the contract was signed the NGO had a legal commitment to implement their project according to the proposal and adhere with financial guidelines. They were reimbursed against quarterly expenditure reports up to the budget agreed at outset. The contracts were renewed annually subject to meeting monitoring requirements and submission of a proposal. A third party evaluation was also carried out at the end of three years. Nothing was stated in the contracts as to what penalties would be imposed if there were a breach of contract, nor what entails a contract breach.

### State X

Established in 1996, XSACS increased the number of NGOs contracted by the state programme from 17 in 1994/1995 to over 100 in 1997/1998 (Seshadri, 2003). There was no historical evidence to provide an indication of the number of NGOs bidding to win the contracts in the original contract rounds and provide an indication of the competitiveness of the market. However, the scale of the programme itself was not sustained. Between 1999 and 2002, the number of NGOs supported fell to between 40 and 65 NGOs, each of them delivering a single project for this funding agency. XMSACS was established in 2001. NGO contracting activities commenced in 2002, with the transfer of all metropolitan based interventions from XSACS to the XMSACS programme.

Once an NGO was recruited and started implementation, claiming funding instalments required the submission of monthly reports for the National AIDS Control Programme (NACP) monitoring system and a report from a monitoring team, consisting of volunteer experts from other NGOs. Contract renewal took place annually with the submission of a proposal for the following year reviewed by a Technical Advisory Committee (National AIDS Control Organisation, 2004). In the event that malpractice was perceived to have occurred, government officials were sent to the NGOs to investigate (XSACS). If the malpractice was proved to have occurred the contract was stopped and the NGO was blacklisted.

XSACS provided 8 different training sessions, attended by 137 NGOs from 1999 to the end of the period of analysis. These were complemented by experience sharing workshops, although these were sporadic (NGO16). There was no uniformity of participation in training or workshops across the NGOs. A single NGO advisor, with support from clerical staff, was responsible for receiving and processing the monitoring and financial reports. Although a post was assigned for a monitoring and evaluation officer, this post was still vacant at the end of the data collection period. The SACS were therefore dependent on the staff of a core group of capable NGOs for carrying out both capacity development and monitoring for the NGO programme.

### State Y

State Y received support for its HIV prevention activities from a number of different sources since 1998. Prior to this, there was little HIV prevention work in the state. In 2001, it was decided that all bilaterally supported HIV/AIDS activities in the state should be channelled through the SACS and the management of all NGO-SACS contracts streamlined and contracted out to one agency. This management agency was awarded the 3-year contract through competitive tender and was paid a pre-agreed fixed annual overhead. It was governed by a project steering committee which meets on a regular basis and comprised representatives from the key stakeholders. The number of NGO contracts rose from 23 in 1999 to 101 in 2002, all delivering a single project for YSACS, except for one which has the responsibility for two projects. Interviews with the management agency revealed that there was a significant competition for these contracts when the work was first initiated, with over 1000 applications made. However, during the second round NGOs were selected from the shortlist made in the first round of recruitment.

The management agency employed a team of full-time experts outside standard government employment conditions, and had the freedom to hire and fire personnel. The budget also allowed for the recruitment of additional agencies to provide further technical support for research, planning and development of educational materials, subject to the approval of YSACS, allowing the programme to draw in extra expertise in a timely manner. As well as providing flexibility, the management agency was a third party to the transaction and therefore able to lobby YSACS on behalf of the NGOs, if necessary, while also increasing transparency in NGO recruitment decisions.

The management agency was intrinsically involved in the NGO contract design and renewal process. Once recruited the NGOs were invited to participate in proposal development workshops and trained in carrying out needs assessment surveys. The proposals were subsequently revised in consultation with the management agency. Contract renewal was a consultative process and again the management agency provides direction in activities, target setting and budgeting. Written guidelines in financial management were also provided to the NGOs to support the contracting process.

The management agency provided a higher quantity of training sessions than in state X, with greater uniformity across the NGOs. In 2001/02 alone they trained 57 batches of NGO staff, covering 13 technical areas. Monitoring visits were also used as a capacity development exercises. If malpractice came to light YSACS requests an investigation which was carried out by the management agency. During the investigation the project was suspended and did not receive funds. Most of the investigations were initiated as a result of anonymous letters or phone calls to YSACS (management agency).

### Institutional environment

Beyond the mechanics of the contract, the study revealed that the institutional environment was characterised by distrust, frequent interruptions in fund flows and weaknesses in capacity for contracting. First, contracting took place in an environment in which there was already significant distrust between NGOs and government and corruption is well known (Bennett & Mills, 1998; Das Gupta, Khaleghian, & Sarwal, 2003; Transparency International, 2002). This distrust was manifest in both the reports of opportunism in the interviews for this study and an imbalance in the contractual relationship. The NGOs did not feel they had many rights within the contract and were vulnerable to frequent delays in the transfer of funds:

“*It is a one-sided document, except to counter-sign the bond… there is nothing legal we can do if they do not deliver…. (we are) powerless in a legal sense to fight funding delays*” (NGO14)

“*They (the NGOs) can’t ask questions – that is the government system*” (XSACS)

Second, the funds released by the National AIDS Control Organisation to the SACS rarely met the budget that had been approved and fund releases were regularly delayed (KI; YSACS). This led to delays in the release of funds to the NGOs. Three NGO case studies experienced delays on 7 different expected releases over 4 years, ranging from a few days to 8 months. NGOs not reporting delays in quantitative form complained that were always delays. In turn this resulted in an inability to plan, in drawing on reserves, the inability to pay salaries, project closures, and an increased level of distrust between the NGOs and their funders. The cause of these delays related to both the expenditure ceiling of the Government of India’s 10th Expenditure Plan and was exacerbated by inadequate resources, financial, human and technical, at the NGO and SACS.

The capacity of the SACS was constrained largely by human resource gaps. During the period of analysis, the SACS in State X had experienced frequent leadership change and empty or inadequately filled staff posts. At XMSACS, during a single year there were four changes of director while the post of NGO advisor remained empty. In addition, decision-making was reported to be constrained by political influence over-riding technical inputs (NGOs 13, 14). As a result XMSACS’ credibility was questioned by the NGOs:

“*They* (the XMSACS technical advisory committee) *check the credibility of the proposal then recommend. But it was not so seriously done. It didn’t give us the impression they had applied their minds at all*” (NGO 14)

Similarly, between 2001 and 2005, XSACS directors remained in post for up to the limited period of 12 months. This was combined with a mixed quality or lack of personnel in posts that are crucial to the function of the programme (NGOs 6, 14, 16). This has weakened the ability to sustain capacity. The NGOs expressed disappointment in the leaders and their frequent changes, stating that programme quality had declined considerably since the early days (KI; NGOs 6, 14, 16).

In contrast, in State Y, there was some continuity of directorship at the SACS level. The director at YSACS prior to the study was in post for a period of 2 years, and was followed by a director who stayed in post for four years. This time allowed for learning as well as a sense of ownership (KI). Although the management agency in State Y was better technically resourced and managed to fill critical NGO programme management posts, a large under-spend in the first year of activity could be partially attributed to the 6 month absence of a Team Leader at the management agency, demonstrating the impact such gaps in human resources can have. In State Y continuity led to capacity development and the facilitating of NGO partnerships, reducing the distrust between the contractual parties.

## Informational problems

### Shared features

Outcomes are difficult to measure in HIV prevention services. The target population is unpredictable and dynamic giving rise to problems in consistent monitoring. This is compounded by the relatively new nature of the HIV/AIDS problem and the low level of awareness even among the NGOs. The training, experience sharing, networking, monitoring and supervision were used to compensate for the highly quantitative emphasis in the formal monitoring system and gather information informally.

### State X

The monitoring system in State X was seen as particularly weak (Dalal Mott MacDonald Pvt Ltd, 2002). Document review of monitoring records at XSACS for other components of this research project (Guinness, Kumaranayake, & Hanson, 2007; Guinness, Kumaranayake, Rajaraman, Sankaranarayanan, Vannela, Raghupathi et al., 2005) found it impossible to track activity and financial reports: there were inconsistent sets of data across NGOs; and complete sets of reports were not available for the majority of NGOs. Quality of the projects was also unknown (Comptroller and Auditor General of India, 2004). External evaluations for State X did not capture quality and NGOs reported that queries from XSACS focused on the financial rather than the technical (NGOs 6.8). Other problems in the system included: lack of feedback from the monitoring teams to the NGOs (NGOs 6,8,19); insufficient resources for NGO supervision (2 KIs); mis-reporting of outputs and activities (XSACS; NGO 6); and potential bias in the monitoring team who were all staff from “competitor” NGOs (other NGOs in contractual partnership with the SACS) (NGOs 6, 8).

“*There is no monitoring system*” (NGO 6).

### State Y

Conversely, in State Y, the NGOs perceived the monitoring as a strength and a vital part of the contracting system (NGOs 2,9), placing importance on its capacity building aspects (NGOs 8,18,19), in spite of the quantitative emphasis (NGOs 9,10,12) and the time required (NGOs 2,15).

“*The monitoring system is best; it allows us to make rectifications… it may be time consuming but it will improve and support our work*” (NGO 18).

The NGOs also placed importance on their relationship with the management agency and YSACS:

“*Transparency is there; ….anything we will discuss; …. Ours* (relationship) *is informal* – *we are friends* ….” (NGO 2).

“*YSACS is a good donor…. providing to the NGOs funds and guidance and training*” (NGO 5).

### Opportunism and ability to control it

Corruption appeared to be an endemic problem. It was present both at the funding agency and NGO levels, comprising bribery, staff exploitation, mis-reporting and the funding of non-existent projects. As can be seen in Table 2, these practises led to an effective reduction in expenditures and activities, a poor reflection of actual activities in monitoring reports, a skewed distribution of budgetary allocations and further mis-reporting. The perceived level of corruption appeared to vary across states and depended on the contracting system. In addition, one key informant criticized XSACS for simplifying its financial monitoring system. Although the former system had caused delays, it had screened out misspending of up to $1 million over three years (KI).

“*Now* (2002) *corruption has come back in*” (KI – on XSACS)

Nevertheless, in spite of concerns regarding corrupt practises, XSACS had blacklisted only one NGO since 1996.

In State Y it appeared that the management agency, as a third party, was able to make NGO recruitment and evaluation more transparent (KI). Facilitating this transparency were the experience sharing workshops where incidence of NGO staff exploitation were uncovered. It appeared better able to overcome opportunistic behaviour:

“…*Can’t give* (external evaluators) *bribes – the NGOs don’t know them*” (NGO 8).

“*There is very little corruption in the* (management agency) *programme*…… (other government funded programmes) *those people they directly ask for money*” (NGO 2).

By mid 2003, the management agency had completed investigations on 9 NGOs and 5 projects had been terminated. In addition, the management agency had used its flexibility to remove their staff when they were found to be involved in bribery (KI).

### Asset specificity

Where asset specificity is present, barriers to entry and exit in the contract are created leaving parties in the contract more vulnerable when opportunism and informational problems arise. The human and temporal asset specific nature of the service led to a dependence on the NGOs on the part of the SACS in both states, although the investment in training, contact time with the NGOs and the development of partnerships with the NGOs varied across the programmes. In State Y, this manifested itself in falling competitiveness in the NGO sector: the second round of NGO recruitment did not involve re-advertisement but a return to the original shortlist of NGO applicants; and, more recently, under the management agency, when a need for re-focussing some of the less successful projects was identified, rather than terminate contracts and re-advertise, the existing NGO partners had their projects re-designed. XSACS presented a concern that the NGOs might seek funding from other sources:

“*If they* (NGOs) *have FCRA certificate* (registered to receive foreign funds) *they will go elsewhere*” (XSACS).

If the NGO lost the contract, there was a real possibility of securing funding from elsewhere given the momentum in HIV programme funding in India at the time. On the other hand, there were also opportunity costs to the NGO of losing the contract, associated with: losing the status of implementing a government supported project; seeking funding elsewhere; and funding gaps that may mean they lose the trust of the communities with whom they work.

### Comparison of the contracting mechanisms in the two states

**Fig. 2:**
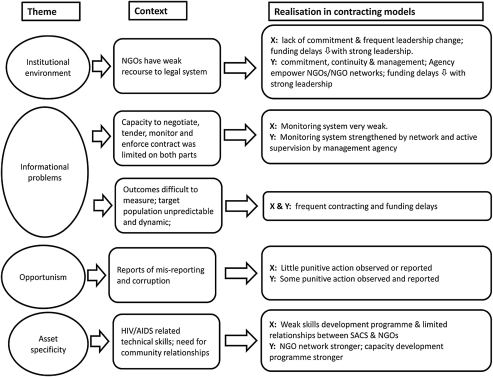
Transaction cost characteristics of the contracting for HIV prevention in states X and Y.

Fig. 2 summarizes the characterization of the transaction costs in the two states. At XSACS the high costs of funding delays and contractual frequency led to the streamlining of financial and other monitoring requirements. Under the conditions of dependence of the funder on the funded, the degree of NGO autonomy and the distance between the NGOs identified in the analysis, state X contracting could be characterized as a relational type governance structure. However, it appears that this structure’s ability to implement the current programme size was limited, with the number of NGO contracts falling during the phase of the programme under analysis. Observations suggest that XSACS simply had too many projects (NGO 6; KI) and inadequate human resources to carry out the intensive monitoring required. Funding delays persisted, problems with meeting monitoring requirements were evident and capacity development had not been sufficiently realised (NGO 6, 16, 14). Added to this, the contracting programme continued to be undermined by opportunism.

YSACS used the management agency mechanism to implement the scale up of its NGO programme. By providing for greater operational independence and flexibility in recruiting technical expertise, the management agency model was able to deliver more NGO projects and scale up the number of contracts faster (Soni & Jani, 2001). The management agency played a directive role in project design and budgeting. All NGOs received training in both technical and managerial aspects of the programme. Capacity development inputs led to strong ties between NGOs and with the management agency, accountability and clarity in the project methods, with one NGO comparing the structure to a company (NGO 15). A technical team strengthened the NGO links with the management agency in a way that the single NGO advisor at XSACS was not able to achieve. However, with the merging of the management structure and as the number of NGOs managed by a single structure had increased, some NGOs found the contractual process declining in quality:

“*With the large number of NGOs now monitoring is not really happening; loose ends are there and misuse*” (NGO9).

“*Now the NGOs are more, the capacity development is less; slowly it has come down. It. is happening but less than compared to before*” (NGO2).

In spite of these problems encountered by the NGOs and persistent opportunistic behaviour, it is evident that the management agency and YSACS still maintained considerable control over the design and implementation of the individual projects and were able to reduce corrupt practises (KI). This greater control and closer relations between the NGOs and the management structure than in state X suggests a more integrated approach to the delivery of the HIV prevention services.

## Discussion

The analysis presented here explores the transaction cost characteristics of two models for contracting out HIV prevention services in India and how closely these relate to transaction cost theory (Williamson, 1987). Using a deductive qualitative approach, based on a combination of document review and semi-structured interviews and supported by the research team’s observations, the analysis shows how transaction costs for these complex contracts appear to be reduced by more hierarchical governance of the NGOs. Paradoxically, however, due to the inflexibility of the Government of India bureaucracy, this form of integration could only be achieved through contracting out the management of the NGO contracts. The Williamson framework was developed for analyses of the private sector and assumes profit maximising behaviour. However, previous analyses in the public sector that build on the Williamson framework have shown that asset specificity, bounded rationality and opportunism are still important factors to consider in the process of contract design (Allen, 2002; Ashton, 1998; Palmer, 2000). In the context of contracting out HIV prevention services in India, where government contracts with not-for-profit organisations, the same factors apply. HIV prevention services are difficult to monitor creating informational problems. There is a need for asset specific investment in training, monitoring and supervision associated with establishing the programme. In addition, the HIV prevention programme in India was plagued with uncertainty arising from fund management and opportunism. According to the theory, the combination of these factors all point towards “serious contractual difficulties” and, in the profit maximizing world, more hierarchical forms of governance. At the same time contracting out of HIV prevention services by the government was a deliberate move away from the Government of India bureaucracy in the search for efficiency.

Hierarchical forms of governance as described by Williamson were not apparent in either of the contractual models under observation in this study. Evidence from both states demonstrates how comprehensive contracting was also not feasible given the combination of bounded rationality and opportunism inherent in the nature of the services, combined with the features of the local context and contract design. XSACS reduced the demands on the monitoring system to minimise delays, depended on a core of technically competent NGOs to help roll out the programme and took a bottom up (NGO led) approach to planning and to contract management. A relational style contract was therefore seen to evolve. The NGOs had freedom to implement their respective project designs and penalties against project mis-management were limited. In addition, the level of technical resources and commitment available at the funding agency has further constrained the ability to effectively manage the contracts. As a result, opportunism and funding delays persisted, there was inadequate investment in capacity development and the number of NGO contracts declined. Without major change in the contracting system and renewed commitment in the form of resources, the success of this relational contracting will be determined by the extent to which opportunistic behaviour of both parties continues.

In State Y, YSACS contracted out NGO contract management to a third party, representing an even greater step away from hierarchical forms of governance. By creating an opportunity for flexibility in management and through the increased availability of resources, the process of contracting out the management of the contracts led to direction in project design, stronger capacity development, greater investment in NGO partnerships and direct supervision. Although the NGOs held a contract with the SACS, they reported to and dealt with the management agency. A relational contract developed between these parties in which opportunism appeared to be reduced by the investment in the NGO-management agency relationship. However, with limited access to the legal system for the NGOs, the power of the SACS to simply stop an NGO contract and the tight supervision of the NGOs by the management agency, the relational contract retained some elements of a hierarchical form of governance. This hybrid form of governance appears to be better able to cope with the informational problems and opportunism identified in the contracting models as well as the scaling up of the NGO programme. Since this study the management agency approach has been rolled out at the national level.

The improved ability to manage the contracts associated with the management agency may have been a result of absolute funding levels rather than the governance arrangements and their ability to minimise transaction costs. Evidence from State X suggests the contrary where, in spite of the budget increasing from INR 40 million to INR 64 million (USD 1 = INR 45.12), XSACS expenditure on the targeted intervention programme fell from INR 55 million (this exceeds the budget as extra resources were allocated to the programme due to its initial success) in 1999/2000 to 26.5 INR million in 2002/03. This implies that the problems did not lie in resource constraints but rather in ability to spend. Although comparable figures are not available in State Y, due to the changing management structures over the period of analysis, it can be inferred that financial costs were necessarily higher with a management agency than without. The analysis suggests that these extra costs helped to offset the opportunity costs of weak monitoring systems, poor technical and financial reporting capacity, NGO distrust of government (and vice versa) and opportunism, even increasing the ability to spend and scale up.

Several limitations to the analysis need to be noted. First, the qualitative nature of the study did not facilitate a direct comparison of the two models and it was not possible to identify on an absolute basis which contracting model minimised transaction costs. Instead, the study aims to identify the nature of those transaction costs and, through analysis of the qualitative information gathered, ascertain which contractual model is more successful in scaling up NGO contracting. Second, the documentation sourced for the study was mixed in quality. There were conflicting figures on expenditures, coverage and number of NGO contracts given in different documents and it was often difficult to discern the reality. Where there was doubt, figures that are reported to National AIDS Control Organisation were used. However, this level of uncertainty in reporting only served to accentuate some of the findings regarding the informational problems. The findings relating to opportunism in state X were also underlined by the lack of communication from NGOs originally selected for our sample. Unwillingness to participate may have stemmed from having something to hide or not having the time. Further enquiries with the key informants suggested that the former was likely in most cases, in which case the analysis has possibly failed to pick up on further evidence of opportunism. With regards to the interviews, many of the NGOs perceived that we came from their funding agencies, in spite of explanations that we were not, and their answers could have been shaped by this perception. In addition, the differences between Indian English and British English complicated the explanation of some issues, possibly leading to misunderstandings or misinterpretation. Further, not all interviews were taped. Although the notes of the interviewer and the research assistant present were cross-checked against each other to minimize these problems, it is recognised that there can be flaws in note taking and they can be more prone to greater subjectivity than taped and transcribed interviews.

As investment in HIV prevention continues and the SACS programmes evolve, the contracting models have important lessons for the future phases of the National AIDS Control Programme as well as to other South Asian countries, where similar NGO contracting programmes are being supported by the World Bank (e.g. Bangladesh and Pakistan). Policy lessons derived from the analysis are threefold. Firstly, contracting out the management of the NGO contracts is a method that can be used to overcome the informational problems associated with the service itself as well as the bureaucratic constraints observed in the SACS. Second, transaction costs might be reduced and project quality improved by re-designing the financial procedures to enable longer term planning, while retaining an incentive for NGOs to perform. Third, an important factor in minimising transaction costs is strong positive relationships between the NGOs and their funder or management agency.

As well as these important policy lessons, the analysis has shown that the transaction cost economics framework provides a useful tool in helping to understand the nature of contractual relations in the public as well as the private sector. It confirms that where uncertainty leads to incomplete contract specification and relational contracting, elements of hierarchical governance that are retained can strengthen the ability of the contract to meet its intended goal and cope with contracting in large numbers.

## Conclusion

The application of the transaction cost framework to the situation of contracting out public services to not-for-profit organisations has allowed the identification of key costs associated with the governance of HIV prevention services through NGO contracts in India. The analysis shows how a relational form of governance with elements of hierarchy evolved within the network of the contract management agency and the NGOs. This has led to greater transparency, improved flows of information and perceived quality, and limited corrupt practises. In turn, the reduction in opportunity costs of poor quality and leakage arising from poorly managed transactions, in spite of higher financial costs, highlights the importance of hybrid models governance systems where full contract specification is not possible and there are “serious contractual difficulties”. It is unlikely that the SACS on its own, with its broader responsibilities and limited autonomy can achieve the same ends. The management agency approach therefore appeared to be both better able to manage large numbers of contracts and transaction cost reducing.

## Figures and Tables

**Table 1:** Description of the State AIDS control societies’ targeted intervention programmes in State X and State Y and the NGO Case Studies, 2002.

	State X	State Y
Programmes	XSACS, XMSACS	Merging of management of state level programmes under one SACS (2001)
Method of contracting	Direct contracting with NGOs	Intermediary carries out contract management, technical support and monitoring and evaluation on behalf of YSACS
Number of NGO targeted HIV prevention projects	XSACS-65, XMSACS-7	101
Of which sex worker projects:	XSACS -21, XMSACS -2	18
No. of case studies	XSACS – 5, XMSACS – 2	11
Of which sex worker projects:	XSACS – 4, XMSACS – 2	9
Average Age of NGO, years (range)	19 (6–38)	18 (5–89)
Average years experience with HIV (range)	7 (4–12)	5 (3–14)
Average annual expenditure of NGO, INR^a^^c^ (range)	3,862,403 (700,000–45,784,404)	2,420,160 (224,800–105,372,462)
Average no. of staff in NGO (range)	43 (8–176)	26 (10–477)
Number of NGOs with FCRA^b^ certificates	5	10

a *N* = 5 for XSACS and XMSACS combined.

b Government issued certificate which allows NGO to receive foreign funds.

c ∗USD 1 = INR 45.12.

**Table 2:** Level and impact of funding delays and corruption on the implementation of the HIV prevention projects.

Problem	Impact on implementation
Mis-direction of funds (NGO 6) Under-appointment and under-payment of staff for whom full salaries are claimed (KI; MA; NGOs 9,10) Mis-reporting of activities (NGOs 6,9,10); mis-use of STI drug and travel allowances (NGO 10; MA) Staff and target group exploitation (NGO 9; MA) Bribing of government officials and management agency staff (XSACS; KIs 1,3,4; NGOs 8,10,14,16,17) Projects funded but not in existence (research team observation)	Effective reductions in expenditure on activities Monitoring does not reflect reality and therefore inability to identify gaps in services Activity levels lower than reported and funded Encourages NGOs to mis-report in order to demonstrate they have met targets Larger budgets awarded to those NGOs willing to pay bribes rather than those with greatest need

MA = management agency.
